# Subcutaneous emphysema and pneumomediastinum in patients with COVID-19 disease; case series from a tertiary care hospital in Pakistan

**DOI:** 10.1017/S095026882100011X

**Published:** 2021-01-20

**Authors:** S. M. Sethi, A. S. Ahmed, S. Hanif, M. Aqeel, A. B. S. Zubairi

**Affiliations:** Department of Medicine, The Aga Khan University Hospital, Karachi, Pakistan

**Keywords:** Coronavirus disease, COVID-19, non-invasive ventilation, pneumomediastinum, subcutaneous emphysema

## Abstract

Since December 2019, the clinical symptoms of coronavirus disease 2019 (COVID-19) and its complications are evolving. As the number of COVID patients requiring positive pressure ventilation is increasing, so is the incidence of subcutaneous emphysema (SE). We report 10 patients of COVID-19, with SE and pneumomediastinum. The mean age of the patients was 59 ± 8 years (range, 23–75). Majority of them were men (80%), and common symptoms were dyspnoea (100%), fever (80%) and cough (80%). None of them had any underlying lung disorder. All patients had acute respiratory distress syndrome on admission, with a median PaO_2_/FiO_2_ ratio of 122.5. Eight out of ten patients had spontaneous pneumomediastinum on their initial chest x-ray in the emergency department. The median duration of assisted ventilation before the development of SE was 5.5 days (interquartile range, 5–10 days). The highest positive end-expiratory pressure (PEEP) was 10 cmH_2_O for patients recieving invasive mechanical ventilation, while 8 cmH_2_O was the average PEEP in patients who had developed subcutaneous emphysema on non-invasive ventilation. All patients received corticosteroids while six also received tocilizumab, and seven received convalescent plasma therapy, respectively. Seven patients died during their hospital stay. All patients either survivor or non-survivor had prolonged hospital stay with an average of 14 days (range 8−25 days). Our findings suggest that it is lung damage secondary to inflammatory response due to COVID-19 triggered by the use of positive pressure ventilation which resulted in this complication. We conclude that the development of spontaneous pneumomediastinum and SE whenever present, is associated with poor outcome in critically ill COVID-19 ARDS patients.

## Introduction

In December 2019, a novel respiratory virus that originated from Wuhan (China), later named as severe acute respiratory syndrome coronavirus 2 (SARS-CoV-2), was found to be the cause of disease called coronavirus disease 2019 (COVID-19). It was recognised as a substantial global public health emergency and SARS-CoV-2 was declared a pandemic on March 11, 2020 [1].

Hypoxaemic respiratory failure leading to acute respiratory distress syndrome (ARDS) is the most frequent complication of COVID-19 [2]. Positive pressure ventilation (PPV) both invasive and non-invasive has proven itself as life-saving rescue treatment for COVID-19 ARDS. PPV use though also has its demerits, with around 1−2% of patients developing barotrauma while recieving it [3]. It occurs due to either increased intra-alveolar pressure, high tidal volume or intrinsic positive end-expiratory pressure (PEEPi), leading to dynamic hyperinflation and is more frequently observed with invasive ventilation compared to non-invasive ventilation [4]. However, in our institute, we observed subcutaneous emphysema (SE) and pneumomediastinum in COVID-19 patients without any exposure to invasive mechanical ventilation, suggesting an alternate pathology.

We identified 10 patients with COVID-19 in our institute who had developed SE and pneumomediastinum while recieving PPV. Nine of them were receiving non-invasive ventilation and one was on invasive ventilation at the time of the event.

We therefore conducted this case series to determine the predisposing factors leading to SE and pneumomediastinum in patients with ARDS associated with COVID-19 disease.

## Methods

### Study setting and duration

Ten patients with COVID-19 who were admitted in the intensive care unit (ICU) of the Aga Khan University Hospital (AKUH), Karachi, Pakistan, from April 2020 till June 2020 were included in the study. The AKUH is a 650-bed, JCIA-certified, tertiary care university hospital, which was the first hospital in the country to admit COVID-19 patients.

#### Study definition

SE and pneumomediastinum occur when air is infiltrated into subcutaneous layers of skin. The extravasation of air to other areas like mediastinum, peritoneum and pleural cavity, is called pneumomediastinum, pneumoperitoneum and pneumothorax, respectively.

*Acute respiratory distress syndrome (ARDS)*: ARDS is an acute diffuse, inflammatory lung injury, leading to increased pulmonary vascular permeability, increased lung weight and loss of aerated lung tissue. It is characterized by hypoxaemia and bilateral radiographic opacities and is associated with increased venous admixture, increased physiological dead space and decreased lung compliance.

As per Berlin's criteria [5], ‘ARDS is defined on basis of bilateral lung opacities appearing within 1 week of clinical insult or new respiratory symptoms, not explained by effusions, collapse or nodules or cardiac failure and fluid overload and having PaO_2_/FiO_2_ ratio less than 300 on a PEEP of < or = 5’.

*Cytokine release syndrome (CRS)*: is a specific form of inflammatory reaction due to the release of pro-inflammatory cytokines, like interleukin-6, in response to infection, certain medicines (chemotherapeutic agent) and other factors. It is graded from 1 to 4 in terms of severity. In the presence of symptoms like fever, nausea, fatigue, headache which are not life threatening, it is graded as 1. In the presence of fluid responsive hypotension or hypotension requiring low-dose vasopressor or oxygen requirement of <40%, it is graded as 2. If oxygen requirement is greater than or equal to 40% or hypotension requires higher dose of vasopressor it is graded as 3 and in the presence of life-threatening features or ventilatory support it is graded as 4 [6]).

#### Inclusion criteria

Patients were included on the basis of laboratory confirmed diagnosis of COVID-19 based on their positive reverse transcriptase-polymerase chain reaction (RT-PCR) assay and chest x-ray (CXR) findings suggestive of SE or pneumothorax or pneumomediastinum.

#### Study design and methodology

This is a retrospective review of clinical data of COVID-19 patients who developed ARDS and then subsequently developed pneumomediastinum and SE. A designed Performa was filled, which recorded demographic data, clinical symptoms, radiological findings, oxygenation status and ventilator requirement and the treatment received, hospital and ICU length of stay and mortality. Duration of positive pressure ventilation and level of positive end expiratory pressure (PEEP), along with cytokine release syndrome grading (CRS grade) at the time of development of SE and pneumomediastinum was recorded.

#### Imaging characteristics

Chest radiology consisted of a standard anteroposterior view. Chest computed tomography (chest CT) was reviewed whenever available. All chest x-ray images were reviewed by a senior consultant radiologist, with greater than 10 years' experience along with their fellow/resident.

The chest radiographs were examined carefully for the presence of pneumomediastinum, SE and pneumothorax. They were also analysed for severity score based on lung opacities at the time of admission and on the day of development of pneumomediastinum or SE. A six-point scoring system was used for objective measurement of lung opacities based on lung zones [7]. Both lungs were divided into the upper, middle and lower zones, and one point was given for opacity in each zone for a maximum score of six.

### Statistical analysis

SPSS Version 23 was used for data analysis. Descriptive statistics was used to summarise the data, and the results were shown in median and interquartile range or mean and standard deviation, as appropriate. Categorical variables were summarised, as counts and percentages.

## Results

We identified 10 patients with COVID-19 from April till June 2020 who met our inclusion criteria. All the patients were treated in ICU although one was not intubated. Three of the patients were admitted directly to the ICU from emergency department (ED) and others were shifted from high dependency unit (HDU) of the hospital. Their demographics and baseline clinical characteristics are shown in Table 1. The mean (±s.d.) age of the patients was 59 (±8) years (range 46–75) and 80% were men. The most common presenting symptom was dyspnoea which was present in all of the patients followed by fever (80%) and cough (80%). The duration of symptoms from the onset until hospital admission was 9.2 days (range 2–20 days). Hypertension was the most common comorbid condition found in six out of 10 patients (60%), followed by diabetes seen in five patients (50%), ischaemic heart disease in three (30%), and one patient (10%) had B-cell lymphoma. The Charlson's comorbidity index ranged from 0 to 4.

**Table 1.** Demographics and baseline patient characteristics

Characteristics	Patient (*n* = 10)
Mean age in years	59 ± 8
(Range)	(46–75)
Gender *n* (%)	
Male	8 (80%)
Female	2 (20%)
Co-morbid *n* (%)	
Diabetes	5 (50%)
Hypertension	6 (60%)
Ischaemic heart disease	2 (20%)
Malignancy	1 (10%)
Mean SOFA score on arrival	2.8 ± 1.0
(Range)	(2–5)
Clinical feature on admission *n* (%)	
Fever	8 (80%)
Cough	8 (80%)
Dyspnoea	10 (100%)
Sore throat	3 (30%)
Bodyache	1 (10%)
Vitals on admission mean ± s.d. (range)	
Systolic blood pressure – mmHg	131.3 ± 19.5 (100–160)
Diastolic blood pressure – mmHg	77.9 ± 8.8 (64–90)
Heart rate – beats per min	106.4 ± 19.0 (88–140)
Respiratory rate – per min	36.9 ± 17.5 (22–80)
Oxygen saturation – %	81.1 ± 12.6 (50–95)

The average of CXR's lung severity scoring was 3.5 out of 6 on admission and 4.35 out of 6, at the time of development of SE. This signifies involvement of greater than 2/3 of lung parenchyma at the time of event (Table 2).

**Table 2.** Demographic and individual patient characteristics

Case	Age (years)	Sex	CCI	Start of symptoms (days)	Type of ventilation	CRS stage	Days of PPV	Total IMV davs	Rx	ICU days	LOH stay (davs)	Outcome	cause
1	75	M	2	NK	NIV	3	4	4	Steroid + T ocilizumab	4	8	Expired	MODS
2	60	M	3	NK	NIV	3	7	7	Steroid + T ocilizumab + plasma	7	19	Expired	MODS
3	56	M	4	NK	NIV	3	2	7	Steroid	7	9	Expired	MODS
4	60	M	2	NK	IMV	3	7(6 = NIV, 1 = IMV)	9	Steroid + T ocilizumab + plasma	9	15	Expired	MODS
5	53	F	5	NK	NIV	3	8	6	Steroid	6	14	Expired	Septic shock
6	49	M	2	7	IMV	4	9	12	Steroid + plasma	12	12	Expired	PTX
7	46	F	2	2	NIV	3	1	9	Steroid + T ocilizumab + plasma	9	19	Sent home	
8	56	M	2	7	NIV	3	6	9(3 + 6)	Steroid + T ocilizumab + plasma	9 (3 + 6)	12	LAMA	
9	65	M	3	NK	NIV	3	8	3	Steroid + T ocilizumab + plasma	3	21	Sent home	
10	65	M	3	11	IMV	4	3	6	Steroid + T ocilizumab	6	17	Expired	GI bleed

CCI, Charlson's co-morbidity index; NK, not known; NIV, non-invasive ventilation; IMV, invasive mechanical ventilation; CRS, cytokine release syndrome; PPV, positive pressure ventilation; MODS, multi-organ dysfunction syndrome; PTX, pneumothorax; LAMA, leaving against medical advise.

At the time of admission all the patients were in hypoxic respiratory failure and their mean PaO_2_/FiO_2_ (P/F) ratio was 122.5 (range 106–145). Two of them had moderate ARDS while P/F ratio of eight was consistent with severe ARDS based on Berlins' criteria. All patients required positive pressure ventilation (PPV), either non-invasive ventilation (NIV) or invasive mechanical ventilation (IMV) since their arrival in the ED.

**Fig. 1.**
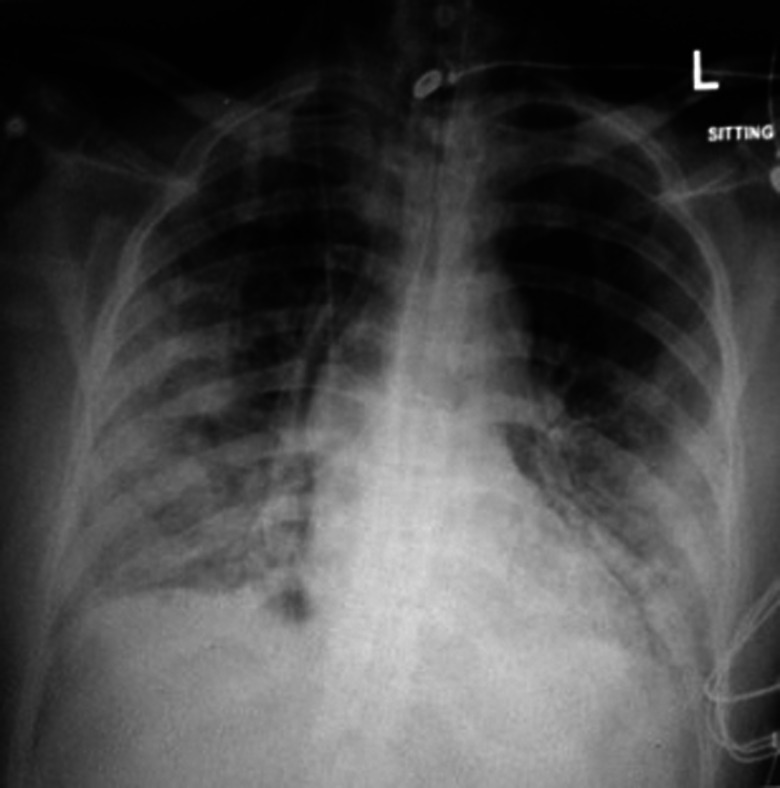
Chest x-ray showing spontaneous pneumomediastinum.

The chest x-ray of eight patients (refer to Table 3) showed spontaneous pneumomediastinum (SPM) at the time of their ED arrival (Fig. 1).

**Table 3.** Imaging findings

No.	PM (initia 1 CXR)	SE (initia1 CXR)	Development of SE in days from DOA	Development of PTX in days	Chest tube placement	Progression to pneumoperi toneum	Intubation (days from positive CXR)	Expired (days from positive CXR)	Cause of expiry	Severity score of CXR on DOA	Severity score of CXR on day of s.e. development
1	Y	N	D3	D3	Y	Y	D4	D10	MODS	3.5	4
2	Y	N	D4	N	N	N	D4	D20	MODS	3.5	4.0
3	N	N	D1 PM, D2 SE	N	N	N	D2	D9	MODS	6	6
4	Y	N	D1	D2	Y	Y	D6	D14	MODS	2.5	3
5	N	N	D1 PM, D2 SE	D8	Y	N	D7	D14	Septic shock	3	4.5
6	Y	N	D4 SE	DIO	Y	N	D2	D14	Tension PTX	5	5
7	Y	N	D2	N	N	N	D2	Sent home	Sent home	4	4
8	Y	N	D1 PM, D3 PM, SE	D3	Y	Y	D11	LAMA	LAMA	4	4
9	Y	N	D14	N	N	N	N	Sent home	Sent home	4/6	4/6
10	Y	N	D1 SE	N	N	N	D6	D20	GI bleed	2.5	5

PM, pneumomediastinum; SE, subcutaneous emphysema; PTX, pneumothorax; CXR, chest x-ray; AXR, abdominal x-ray; DOA, date of admission; MODS, multi-organ dysfunction syndrome; LAMA, left against medical advise.

**Fig. 2.**
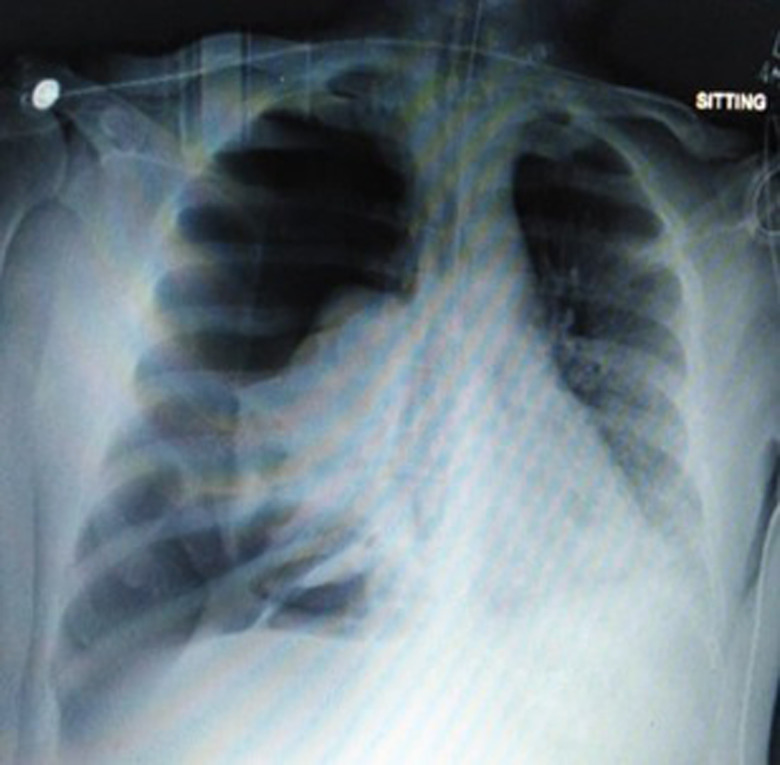
Chest x-ray showing right pneumothorax.

The other two patients developed it after 1 day of ED stay. With the progression of time, all of the patients developed SE after days 1–4 of developing SPM. One patient developed it after day 14 of SPM. Five of the patients also developed pneumothoraces. One patient's CXR showed SE and pneumothorax developing on the same day (Fig. 2).

**Fig. 3.**
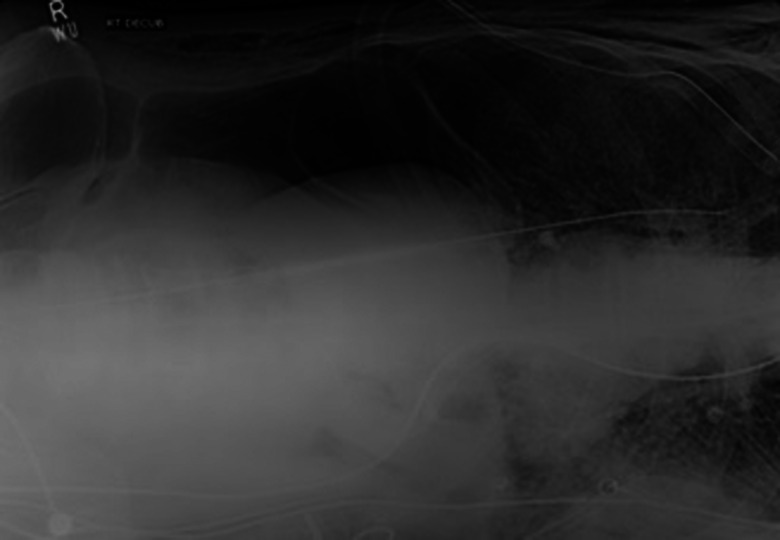
Abdominal x-ray showing extensive pneumoperitoneum.

All others develop it after 3–10 days of developing SE. Three patients also had an extension of air into peritoneum as seen by their abdominal x-ray (AXR) and computerised tomography (CT) scan (Fig. 3).

The median duration of assisted ventilation before SE ranged from 1 to 9 days (average 5.5 days). Seven of the patients developed it while recieving NIV and three were on invasive mechanical ventilation at the time of SE. The average PEEP at that time, for those on NIV was 8 cmH_2_O (range: 8–16 cmH_2_O) and for those on invasive ventilator was 10 cmH_2_O (range: 8–12 cmH_2_O). Five patients had chest tube insertion and others were managed conservatively. All patients required endotracheal intubation except for one who was managed initially on non-invasive ventilation and then face mask. Invasive mechanical ventilation was required either on the same day or second day of development of SE. The average length of ICU stay was 8.6 days (range 3–14 days).

At the time of development of SE, all patients were found to have CRS. Eight patients were in CRS grade 3. Two were in CRS grade 4 and both of them expired. The median ferritin level for the group was 2354.5 ng/ml (interquartile range, 1156−4427), the median lactate dehydrogenase (LDH) level was 868.5 IU/l (interquartile range, 767.5−1188.7), and the median C-reactive protein (CRP) level was 229 mg/l (interquartile range, 116.7−345.5). Creatinine phosphokinase (CPK) level of only one patient was checked which was 2632 IU/l (normal range: 46–171 IU/l).

All patients in our study received steroids, and five patients received a combination of tocilizumab and convalescent plasma along with steroids. The only two survivors in our case series received this combination therapy. Seven patients (70%) received tocilizumab either with plasma or with steroids and six patients received convalescent plasma. All the patients except one underwent prone positioning, either conscious proning or on mechanical ventilator. Central venous catheter (CVC) were inserted in seven patients. Out of those three had CVC insertion after pneumomediastinum and SE and four had CVC insertion prior to these complications and had developed subcutaneous emphysema on post insertion day 1, 2, 3 and 10 respectively.

Out of the 10 patients studied, seven expired, two of them survived and one left against medical advice (LAMA). Out of the seven non-survivors, pneumothorax and pneumomediastinum leading to obstructive shock, was the cause of death in only one patient. Five of the non-survivors, developed multi-organ dysfunction syndrome (MODS) and septic shock and one, developed lower GI bleed after successful extubation and complete resolution of SE. The two survivors had conservative management of their pneumomediastinum without chest tube insertion, although they received combination treatment with steroids, tocilizumab and convalescent plasma. One of the survivor was never intubated and was managed only on NIV. All patients in our case series, both survivors and non-survivors had a prolonged hospital stay with an average of 14 days (range 8−25 days).

## Discussion

This is the first case series on clinical characteristics and outcomes of SE and pneumomediastinum in COVID-19 patients with ARDS from Pakistan. Pneumomediastinum and SE usually occurs after rupture of an over distended alveolus with air leaking into surrounding mediastinum and along cervical fascial planes into subcutaneous tissue. It can lead to pneumothorax, which is defined as the presence of air between parietal and visceral pleura causing difficulty with oxygenation [4]. The causes of pneumomediastinum, SE and pneumothorax can be spontaneous or traumatic.

It can also be a complication of positive pressure ventilation. We have observed increased frequency of these complications in COVID-19 ARDS patients compared to ARDS due to other causes. Our patients were mostly male with mean age of 59, which is the age group (50–70) associated with most critical course of COVID-19 in our country [8, 9]. Diabetes and hypertension were the most common comorbid conditions, which coincides with COVID-19 dataset generated by the CDC from the 13 US states [10]. None of our patients were smokers or had any underlying chronic lung condition prior to the illness.

As all of the patients in our case series were on positive pressure ventilation at the time of the development of SE, barotrauma can be hypothesised as a cause of this complication. Seven of our patients were on non-invasive ventilation and three were on invasive mechanical ventilation. Barotrauma manifesting as pneumomediastinum, SE and/or pneumothorax is a known complication of positive pressure ventilation. It is usually a form of ventilator-induced lung injury (VILI) occurring due to increased stress and pressure in alveoli. The susceptibility of lungs to develop VILI is non-homogenous with some regions more at risk of barotrauma than others. Dependent part of the lung can be damaged by shear forces required for cyclical alveolar collapse and reopening during expiration and inspiration with insufficient PEEP. Similarly, non-dependent parts of the lung are more susceptible to rupture with high inspiratory pressure of tidal volume. This complication is though more reported with invasive ventilation than non-invasive ventilation. Low tidal volume and low plateau pressure strategy is therefore in practice in managing ARDS patients on invasive mechanical ventilation to protect lung from this complication [11]. Seventy per cent of our patients though were on NIV including BIPAP and CPAP, at the time of development of SE (Tables 4 and 5).

**Table 4.** Laboratory and radiological investigations

Laboratory data	Patient (*n* = 10)
On admission: median (IQR)	
White cell counts – × 10^9^/l	9.05 (5.5–13.6)
Platelets – × 10^9^/l	261.5 (183–357)
Serum creatinine – mg/dl	1.1 (0.7–1.7)
Total bilirubin – mg/dl	0.7 (0.4–0.9)
Infective Markers: median (IQR)	
Highest ferritin – ng/ml	2354.5 (1156–4427)
Highest LDH – IU/l	868.5 (767.5–1188.7)
Highest CRP – mg/l	229 (116.7–345.5)
Highest procalcitonin – ng/ml	0.57 (0.36–6.29)
Highest D-dimer – mg/l FEU	5.35 (1.8–13.4)
pH: median (IQR)	
On admission	7.43 (7.35–7.47)
Before pneumomediastinum	7.42 (7.40–7.51)
pO_2_: median (IQR)	
On admission	71.7 (57.7–80.2)
Before pneumomediastinum	59.5 (51.9–65)
pO_2_/FiO_2_ ratio: median (IQR)	
On admission	122.5 (106–145)
Before pneumomediastinum	71.5 (53–97)
Imaging: *n* (%)	
Subcutaneous emphysema	8 (80%)
Pneumomediastinum	10 (100%)
Pneumothorax	5 (50%)
Pneumoperitoneum	3 (30%)

**Table 5.** ARDS categorisation, treatment, assisted ventilation and clinical outcomes

	Patient (*n* = 10)
ARDS: *n* (%)	
Moderate – (PaO_2_/FiO_2_ = 100–200)	2 (20%)
Severe – (PaO_2_/FiO_2_ < 100)	8 (80%)
Treatment: *n* (%)	
Steroid	10 (100%)
Tociluzumab	6 (60%)
Convalescent plasma	7 (70%)
Proning: *n* (%)	
Yes	9 (90%)
No	1 (10%)
Ventilation: *n* (%)	
Non-invasive mechanical ventilation	7(70%)
Invasive mechanical ventilation	3 (30%)
Clinical outcomes:	
Median duration of assisted ventilation before pneumomediastinum (IQR) – days	5.5(1–9)
Median length of hospital	14 (8–25)
Length of ICU stay	8.6 (3–14)
Chest tube placement *n* (%)	5 (50%)
Mortality *n* (%)	7 (70%)
Discharged home *n* (%)	3 (30%)

Despite frequent use of NIV for acute respiratory failure due to varied causes, SE is very seldom reported with its use in literature [12, 13] before COVID-19. It is therefore postulated that COVID-19 patients with extensive lung damage might have increased respiratory drive with persistent strong spontaneous inspiratory efforts causing self-inflicted lung injury as postulated by Gattinoni [14]. End expiratory pressure provided by NIV increases the pressure gradient between alveoli and interstitium. This pressure gradient might have over distended, already damaged alveoli causing their rupture with further extension of air into mediastinum and the pleura and subcutaneous tissue.

Another important finding to support this hypothesis is that at time of development of SE, all of the patients, were either in grade 3 or grade 4 CRS, signifying severe inflammation process (Table 2).

Radiologically extensive lung involvement was also seen in these patients's CXR. CXR's lung severity score averaged 3.5 out of 6 on admission and 4.35 out of 6, at time of development of SE, signifying involvement of greater than 2/3rd of lung parenchyma. This further supports our postulation that in COVID-19 ARDS, severe lung damage with concomitant rise in intra-alveolar pressure might be a cause of spontaneous rupture of these hyper-inflated alveoli with dissection of air along the bronchovesicular sheath into mediastinum, pleural cavity and subcutaneous tissues (Macklin effect). This progressive development of pulmonary interstitial emphysema (PIE) has been experienced in a variety of other viral pneumonias and also in patients with severe underlying lung disorder which affects alveoli (like ARDS, COPD, necrotising lung parenchymal infection, influenza bronchiolitis, *Pneumocystis carnii* pneumonia and even severe acute respiratory syndrome (SARS)) [14]. Applying current understanding of the disease to the existing knowledge it can be said that SARS-CoV-2 virus might cause a similar degree of lung damage making alveoli more susceptible to rupture.

This postulation of COVID-19, being a disease associated with diffuse alveolar damage like SARS is further supported by the fact that 8/10 of our patients had spontaneous pneumomediastinum on their arrival CXR and the remaining two patients developed it on second day of admission. The presence of spontaneous pulmonary air leak on admission, before application of positive pressure ventilation is signifying an alternative pathology other than barotrauma for occurrence of this complication in COVID-19 ARDS patients.

Similar findings of spontaneous pneumomediastinum is also reported in three other published case reports about COVID-19 patients [3, 15, 16].

**Fig. 4.**
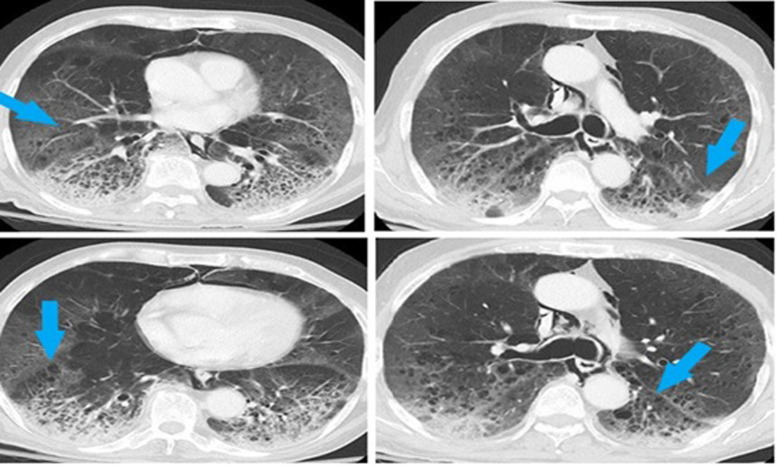
CT chest showing ground glass haziness with pulmonary cysts.

One of our patient's computed tomography of the chest showed ground glass haziness and opacities in bilateral lungs along with multiple randomly scattered pulmonary cysts of variable sizes bilaterally, more marked in lower lung zones (Fig. 4).

A case report from Wuhan also described multiple bullae in the computed tomography of the chest in a patient developing SE [17]. These bullae could be an atypical presentation of COVID-19 with tendency to rupture spontaneously. Lung condition like *Pneumocystis carinii* pneumonia are known to cause lung cysts and pneumatocoele with tendency to spontaneously rupture [18]. Coughing is a common symptom of COVID-19 and was also present in eight out of 10 of our patients. Frequent bouts of cough can be a critical factor contributing to this complication. It can be presumed that NIV application during bouts of coughing might have caused rapid increase in the peak inspiratory pressure with resultant alveolar septal rupture. It is observed in past that in the presence of peri-bronchial fibrosis, NIV application particularly during coughing attack might increase traction on small airways causing discontinuation of bronchoalveolar junction [19].

**Fig. 5.**
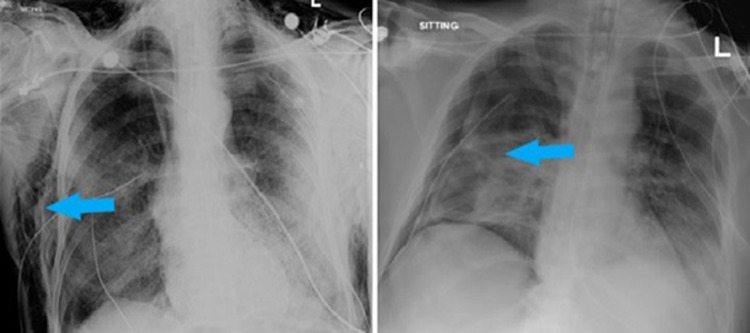
Chest x-ray showing SE (right image) and pneumothorax with chest tube on right side (left image).

**Fig. 6.**
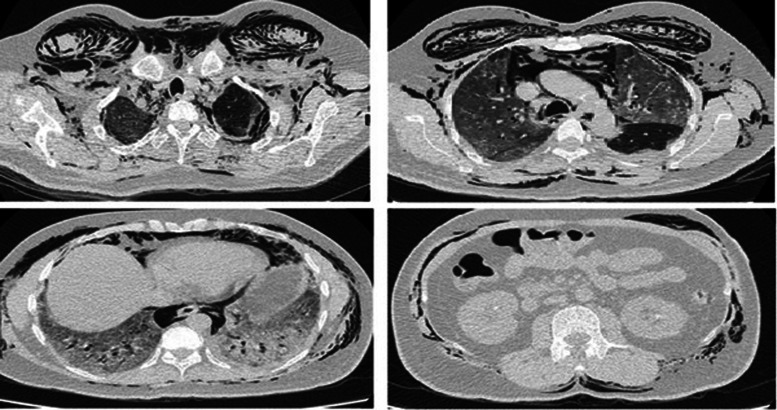
CT chest and abdomen showing extensive SE and pneumoperitoneum.

Of note our results show that in all of our patients spontaneous pneumomediastinum occurred first and then SE developed later over a period of 1–5 days. In five cases it progressed to pneumothorax and in three cases air dissected even into lower half of the body causing pneumoperitoneum (Fig. 6).

The outcomes were poor in majority of our cohort with 9 out of 10 patients undergoing intubation and seven of them expired. It seems like development of spontaneous pneumomediastinum and SE which on their own are benign complications, are associated with worse prognosis in COVID-19 patients. As the recorded mortality in COVID-19 patients receiving mechanical ventilation in our institute is around 25–30%, this signifies development of spontaneous SE as a strong predictor of mortality in these patients. Studies would be needed in future to look into prognostic implications of these findings.

The two patients who survived in our case series had received conservative treatment without any tube thoracostomy. Five of our patients who developed worsening pneumomediastinum, pneumothorax and haemodynamic compromise, underwent chest tube thoracostomy. None of them survived.

Five of our patients received a combination therapy of steroids, convalescent plasma and tocilizumab. Two of the survivors received the combination treatment along with other ICU management. As the sample size is small, and there are many confounders which were not taken care of because of the descriptive nature of the study, no inference about the treatment can be drawn.

Retrospective collection of data is a major limitation of our study due to which we might have missed some information regarding clinical and ventilator parameters. The other limitations were small sample size, actively evolving treatment protocols of a new disease, observational case series from a single tertiary care centre. Four patients had central line insertion before development of SE but only one patient developed it on the next day. Dissection from CVC insertion might have introduced external air and have confounded the picture. One major limitation of our study is the scoring system used to measure opacities based on lung zone. Despite simplicity of use this CXR scoring system is only experimental and would need further validation study to be taken as standard. An additional limitation was the absence of CT scan chest in majority of patients which would have clearly defined the lesion and Macklin effect, if any.

However despite these limitations, this study adds to existing knowledge about COVID-19 and would help in designing future studies to look into this complication further.

## Conclusion

Our findings suggest that it is lung damage secondary to inflammatory response due to COVID-19 triggered by the use of positive pressure ventilation which resulted in this complication. We conclude that although development of spontaneous pneumomediastinum and SE are rare complications in critically ill COVID-19 ARDS patients but are associated with worse prognosis.

## Data Availability

Our findings are conceptual/theoretical and does not require any data statement.
